# Clinical classification and treatment of cubital tunnel syndrome

**DOI:** 10.3892/etm.2014.1983

**Published:** 2014-09-22

**Authors:** CUI QING, JIANHUA ZHANG, SHIDONG WU, ZHAO LING, SHUANCHI WANG, HAORAN LI, HAIQING LI

**Affiliations:** Department of Orthopaedics, Cangzhou Hospital of Integrated Traditional Chinese and Western Medicine, Hebei Medical University, Cangzhou, Hebei 061001, P.R. China

**Keywords:** classification, treatment, cubital tunnel syndrome

## Abstract

The aim of the present study was to investigate a new clinical classification of cubital tunnel syndrome that provides an improved basis for the clinical diagnosis and treatment of the disease. Retrospective analysis was performed on 341 patients with cubital tunnel syndrome. Based on the etiology, signs and symptoms, neurophysiological tests and computed tomography (CT) imaging, a new clinical classification was proposed. The patients enrolled in the study were treated according to the new classification. According to the new classification, cubital tunnel syndrome cases were divided into types I-IV. Treatment for patients with type I consisted of rest, immobilization or physiotherapy, while patients with type II received simple ulnar neurolysis. Type III patients underwent ulnar neurolysis with expansion of the ulnar nerve sulcus or ulnar nerve anterior transposition surgery. Type IV patients represented a subgroup of cubital tunnel syndrome cases caused by factors other than degenerative joint diseases, including cysts, tumors, traumatic fracture, deformity and elbow deformity. Patients of this type received appropriate surgical treatment according to the specific etiology. Based on previous classifications that relied on sensation and strength symptoms, a new clinical classification of elbow tunnel syndrome has been established in the present study that adopts a CT imaging evaluation index. The new classification is reasonable, simple and practical, and therapies based on this classification are more targeted than those based on previous classifications.

## Introduction

Cubital tunnel syndrome, or ulnar nerve entrapment syndrome, is also known as tardive ulnar neuritis (1,2). The cubital tunnel is the most common site for entrapment in this syndrome (3). Previous studies have formed clinical classifications of cubital tunnel syndrome based on sensation, movement and elbow flexion tests or Tinel’s sign (4–6). These classifications rely only on subjective symptoms and objective signs and lack quantitative indicators. On the basis of these classifications, Gu (7) proposed a new classification that includes neurophysiological tests as a diagnostic quantitative index for cubital tunnel syndrome. Compared with other clinical classifications and treatment programs, Gu’s system adopts electromyography (EMG), an internationally recognized diagnostic index for cubital tunnel syndrome. The system is based on quantitative neurophysiological indicators, as shown in Table I. According to Gu’s classification, patients with cubital tunnel syndrome may be divided into three types: Mild, moderate and severe. Patients classified as moderate are recommended to receive neurolysis decompression surgery, whereas patients classified as severe should be treated with anterior transposition.

However, clinical diagnosis and treatment efficacy when using this classification system may be unsatisfactory in certain patients with elbow osteoarthritis, elbow deformity or cubital tunnel mass oppressors. According to Gu’s classification, these patients are classified as mild or moderate and should receive conservative or neurolysis treatment. However, we hypothesize that for patients with deformities of the elbow or cubital tunnel tumors, treatment should not be based on the previous classification as it lacks radiographic evaluation of the elbow structures. Instead, these patients should be classified separately and receive targeted therapy based on a combination of ultrasound, imaging and other tests.

Therefore, the aim of the present study was to develop a new classification for cubital tunnel syndrome to further improve clinical treatment.

## Patients and methods

### Patients

Between December 2002 and March 2011, a retrospective analysis was performed on 341 patients diagnosed with cubital tunnel syndrome in the outpatient and inpatient departments of Cangzhou Traditional Chinese and Western Medicine Hospital (Cangzhou, China). The patients included 279 male and 62 female cases, aged between 16 and 79 years (mean, 51.3±10.6 years). In 22 cases, the disease was present in both elbows. With regard to occupation, 109 cases were construction workers, 91 patients were farmers, 105 cases were handicraft workers, 13 individuals were students, 9 patients were public officials and 14 cases had other occupations. There were 253 patients with elbow osteoarthritis, accounting for 74% of the cases. Patients were evaluated using the cubital tunnel syndrome function impairment indexing method developed by Goldberg *et al* (8). This assessed subjective symptoms, including numbness or tingling, the two point discrimination test and interosseous muscle strength. Cubital tunnel syndrome was diagnosed using the following diagnostic criteria: Hypersensitivity, diminished sense or a loss of sense of the dorsal ulnar palm and ulnar half of the ring and little fingers, which may be accompanied by hand intrinsic muscle atrophy, weakness or claw hand deformity. Secondly, the presence of a positive Tinel’s sign and, finally, abnormal sensory and motor nerve conduction velocity at the elbow. All patients underwent neurophysiological (EMG and sensory and motor nerve conduction velocity measurements) and X-ray examinations and 78 patients underwent computed tomography (CT) examinations. The cubital tunnel index was determined based on elbow CT scans combined with the aforementioned classifications (9).

**Figure 1:**
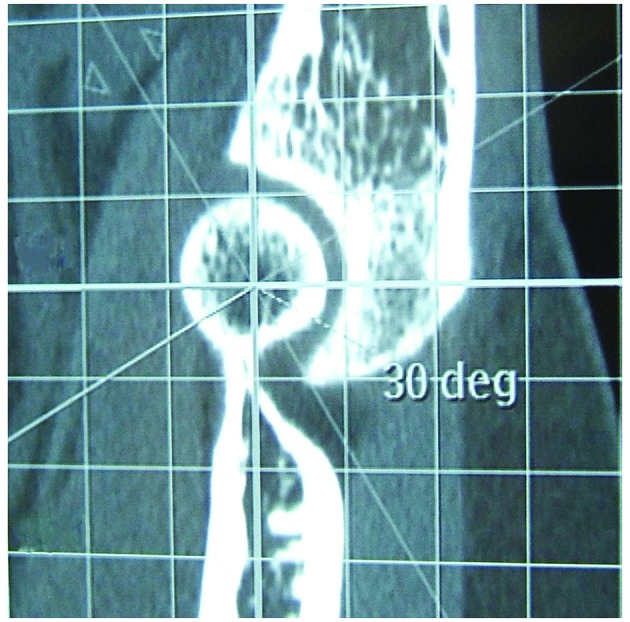
Positioning of a normal adult cubital tunnel at 30° for a flat CT scan. CT, computed tomography.

**Figure 2:**
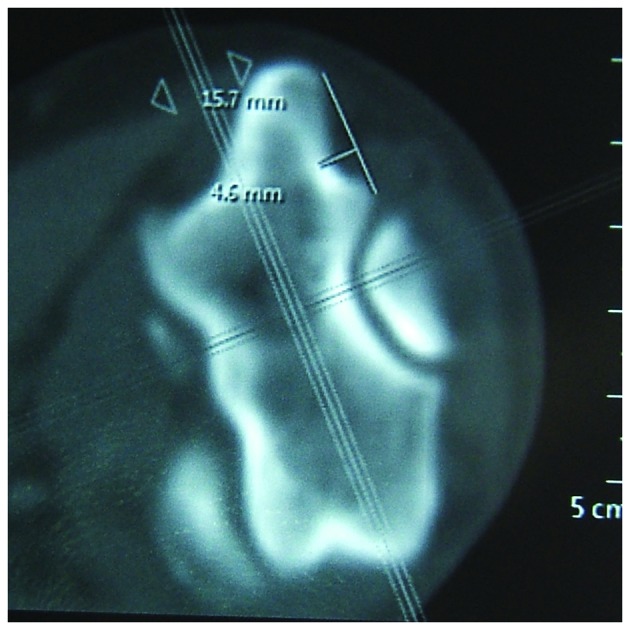
Normal adult cubital tunnel at 30° for a flat CT scan. CT, computed tomography.

The specific calculation method for the cubital tunnel index was as follows: Over the Hueter line (medial-lateral condyle connection), the cross-section of the humeral shaft was rotated forward 30° for the CT scan to measure the depth and width of the elbows in the cross-section. The depth/width ratio was calculated and defined as the cubital tunnel index. An additional 102 healthy adult volunteers (male, 89; female, 13; age, 21–52 years; mean, 32.4±4.5 years) were recruited as controls. Over the Hueter line, the vertical cross-section of the humeral shaft was rotated forward 0, 15 and 30° for plain CT scans. The depth and width of the ulnar nerve sulcus on the corresponding cross section were measured (Figs. 1 and 2). The study was approved by the Ethics Committee of Cangzhou Hospital of Integrated Traditional Chinese and Western Medicine and all the healthy volunteers and patients provided informed consent.

### Statistical analysis

According to the measured depth and width of the ulnar nerve sulcus, the depth/width ratio was calculated and presented as mean ± SD. SPSS software, version 13.0 (SPSS, Inc., Chicago, IL, USA) was used to conduct paired t-tests. P<0.05 was considered to indicate a statistically significant difference. There was no significant difference in depth, width or the depth/width ratio in healthy controls at 0°, 15° or 30° between the left or right elbows (Table II). Therefore, the mean values of the two elbows at 0, 15 or 30° were combined and compared with the values of the patients with cubital tunnel syndrome.

## Results

### Healthy controls

In the healthy volunteers, at 0°, the cubital tunnel depth was 1.780±0.669 mm and the width was 12.907±2.226 mm. Thus, the depth/width ratio was 0.130±0.044. At 15°, the cubital tunnel depth was 2.741±0.831 mm, the width was 13.443±2.611 mm and the depth/width ratio was 0.204±0.056. At 30°, the cubital tunnel depth was 3.590±0.886 mm, the width was 13.271±2.409 mm and the depth/width ratio was 0.273±0.055.

### Patient classification

Patients with Type III cubital tunnel syndrome were classified according to the Dellon/Gu classification standard and 59 cases were identified as mild, 156 cases were moderate and 38 cases were considered to be severe. In 21 mild cases receiving conservative treatment, the symptoms worsened progressively over the 6-month treatment period. CT imaging revealed the existence of osteoarthritis in the ulnar nerve sulcus and an abnormal cubital tunnel index. Thus, cubital tunnel expansion was performed in these patients.

Based on the etiology, symptoms and signs, neurophysiological tests and CT imaging, patients with cubital tunnel syndrome were divided into types I-IV (Tables III and IV). There were 27 type I cases that experienced ring and little finger numbness, weakness, incoordination and ulnar nerve irritation/aggravation when the elbow was flexed. These individuals had a positive Tinel’s sign, but exhibited normal neurophysiological function and had a normal CT cubital tunnel index. A total of 49 patients were classified as type II and they exhibited ring and little finger numbness, a positive Tinel’s sign, poor grip strength and decreased interosseous muscle strength, with or without atrophy. Neurophysiological tests revealed reduced motor and sensory nerve conduction velocity, but the cubital tunnel index was normal. There were 253 patients classified as type III. These individuals exhibited ring and little finger numbness, a positive Tinel’s sign, poor grip strength, interosseous muscle strength loss and muscle atrophy. In addition, nerve electrophysiological examinations revealed reduced motor and sensory ulnar nerve conduction velocity and the cubital tunnel index increased or decreased. A total of 12 patients had type IV cubital tunnel syndrome and experienced ring and little finger numbness, a positive Tinel’s sign and neurophysiological tests showing reduced ulnar nerve elbow motor and sensory conduction velocity. CT imaging revealed the patients to have a normal cubital tunnel index, while elbow ultrasound, CT or magnetic resonance imaging (MRI) examinations revealed cysts, tumors, other non-degenerative diseases or elbow deformities.

### Treatment

Treatment for type I patients was rest and physical therapy, while type II patients received simple ulnar neurolysis. Type III patients received ulnar neurolysis plus expansion of the ulnar nerve sulcus or ulnar nerve anterior transposition surgery, according to the common approach for the treatment of cubital tunnel syndrome (10). Among the type III patients, increases in the cubital tunnel index were observed in patients with primary elbow osteoarthritis, osteophytosis and entrapment of the ulnar nerve caused by narrowing of the ulnar nerve sulcus. Type IV patients underwent surgical removal of any cyst or tumor where compression was caused by the cysts or tumors. Patients with elbow valgus deformity received orthopedic surgery or simultaneous ulnar neurolysis.

For ulnar neurolysis, special attention was paid to the entrance and exit of the cubital tunnel. If entrapment occurred, it was released completely and a cubital tunnel expansion was performed (11). In the present study, three patients exhibited elbow valgus deformity and five patients had post-traumatic scarring of the surrounding tissues of the nerve. These five patients underwent ulnar neurolysis, which included the release of the aponeurosis at the entrance and exit of the cubital tunnel. In the three patients with an elbow valgus deformity, cubital tunnel expansion was performed in addition to ulnar neurolysis including the release of the aponeurosis at the entrance and exit of the cubital tunnel. The advantages were that the cubital tunnel walls expanded and deepened, causing the cubital tunnel volume to increase without changing the normal anatomy of the ulnar nerve structure or route. The surgery involved an elbow medial incision that was 5–6 cm in length. Firstly, the arcuate ligament was isolated from the humeral condyle and protected. It was important to protect the articular branch blood vessels when releasing the compressed ulnar nerve. The periosteum of the ulnar nerve sulcus was peeled from one side and repaired thinly. Using a high-speed burr, the ulnar nerve sulcus was expanded to 8×10×25–30 mm. The periosteum was then sewn back into position and the ulnar nerve was placed back into the reformed ulnar nerve sulcus. The arcuate ligament was also sewn back into the original position. For cubital tunnel expansion or simple neurolysis, the proximal and distal ends of the cubital tunnel were conventionally explored and released.

### Outcomes

All patients in the study were treated according to the new clinical classifications aforementioned. Satisfactory results were obtained when evaluated by the efficacy grading system described by Lascar and Laulan (10). A total of 314 patients underwent surgery and the patients were followed-up for 3–12 months following surgery, with an average follow-up time of 7.3 months. In total, 13 patients that underwent simple ulnar neurolysis required repeated surgery, including 12 patients with elbow osteoarthritis and one patient with post-traumatic elbow deformity. Four patients underwent cubital tunnel expansion and nine patients underwent ulnar nerve anterior transposition. All the patients experienced improved postoperative numbness and muscle weakness and the postoperative results were satisfactory. At the last follow-up, 137 patients underwent neural electrophysiological examinations and the results demonstrated that nerve conduction velocity improved by 92% compared with the preoperative status. Key-pinch power was improved in 365 patients, with the average increasing between 53.1 and 78.9% following surgery. However, in nine patients with severe preoperative interosseous muscle atrophy, no improvement in the key-pinch power was observed at the final follow-up.

## Discussion

The new classification of cubital tunnel syndrome presented in the current study is based on previous classification systems. Etiology, sensation, movement, neurophysiological observations and the cubital tunnel index, obtained from CT imaging, are analyzed comprehensively. Based on the corresponding quantitative indicators, cubital tunnel syndrome is classified, providing the basis of clinical treatment for each type. The classification system is simple, practical and provides a basis for treatment. In addition, the surgical procedures are well targeted, which has great guiding significance in the clinical diagnosis and treatment of cubital tunnel syndrome.

In the present retrospective study, the occupations of the patients were mainly heavy manual workers, including construction workers, farmers and craftsmen (mainly metal pipe manufacturing workers), which may have been the cause for the high percentage of patients with elbow osteoarthritis. The incidence of elbow osteoarthritis was 74%, which was significantly higher compared with the incidence rate of 2% reported by a mass epidemiological study (12). A possible reason may be that the patients in the present study suffered from elbow diseases, which caused the incidence of elbow osteoarthritis to be higher than that observed in the general population. A high incidence of elbow osteoarthritis was also observed in the study by Kato *et al* (13) where 472 cases (487 elbows) were analyzed retrospectively and the elbow osteoarthritis prevalence was 64%. Among the 38 cases with medial elbow ganglia lesions, 37 cases exhibited degenerative changes, as shown by X-ray examination (13).

Comparisons among X-ray, CT and MRI scans of cubital tunnel structures indicate that X-ray radiography is frequently affected by artificial factors and it does not clearly distinguish the cubital tunnel structure. MRI is an option, but it provides low resolution imaging of the cubital tunnel structures at a high cost. By contrast, CT scans show clear imaging of the structures in the cubital tunnel with accurate positioning and good repeatability. This is particularly important for preoperative assessment and operational planning in type II and III cases, particularly type III patients who require elbow laminoplasty or ulnar nerve anterior transposition. For these reasons, the CT cubital tunnel index should be regarded as an important index for classification. CT examinations further clarify the bony change of the cubital tunnel, and cubital tunnel expansion may be performed for bony compression of the cubital tunnel (14,15). CT examinations are not required for every patient. For the majority of patients without elbow deformity or limited motion, CT is not required. It is more suitable for patients with osteoarthritis, post-traumatic elbow lesions, tumors or for patients that require secondary surgery or cubital tunnel expansion.

Compared with CT, ultrasound examination has a higher sensitivity to soft tissues, but has a poorer ability to identify bone tissues. It has been hypothesized that the ability of ultrasound to visualize nerves may prove to be useful in cases of peripheral nerve trauma, tumors or revision surgery (16–18). In addition, ultrasound is able to detect structural changes in the soft tissues surrounding the cubital tunnel, neuropathy and tumors. However, human factors play a significant role in ultrasound examination. Different ultrasound technicians may produce significantly various ultrasound results for the same patient. In China, no orthopedic surgeon performs ultrasound examination themselves, which is why orthopedic surgeons are not likely to determine a surgical plan based on ultrasound results solely when other clearer and more precise imaging results are available.

In the present study, over the Hueter line, the cross-section of the humeral shaft (0°) was rotated 30° forward for CT imaging. The corresponding depth and width of the cross-section were measured for the calculation of the cubital tunnel index, defined as the ratio of the depth and width. The reasons for this are that at the elbow, the humerus is flat, wide and curled forward. It has 30–50° anteversion of the humeral long axis. The distal humerus widens at its two ends, forming the medial epicondyle. With accurate positioning, the Hueter line within the epicondyle is observed. According to the morphological observations and plain CT scans of the cubital tunnels, in the cross-section over the Hueter line, the depth of the cubital tunnel was found to increase gradually between the proximal and distal ends. The ulnar nerve sulcus was shallow in specific patients and thus invisible in the 0 and 15° cross-sections. In these cases, it was not possible to measure the depth and width of the cubital tunnel.

In patients with elbow osteoarthritis, the closer the osteoarthritis to the joints, the more marked the hyperosteogeny. At the center of the medial epicondyle, 45° distal rotation exactly reaches the export position of the cubital tunnel. Rotation 30° forward is within the cubital tunnel and consistent with the anatomical characteristics that the lower end of the humerus curls 30–50° forward. Thus, the depth/width ratio of the cubital tunnel measured using the cross-section with forward rotation of 30° was defined as the cubital tunnel index and used for clinical classification in the present study.

For patients with a decreased cubital tunnel index, shallow ulnar nerve sulcus, slippage of the ulnar nerve, increased cubital tunnel index and narrowing of the cubital tunnel due to hyperostosis, ulnar sulcus expansion or ulnar nerve anterior transposition should be performed. For those with a normal cubital tunnel index, elbow depth and width, cubital tunnel syndrome primarily results from compression by a tumor or soft tissue edema. Ulnar neurolysis is indicated for these patients. This procedure solves the issue that when the elbow flexes, the cubital tunnel volume becomes smaller, the pressure increases and the ulnar nerve is stretched and potentially injured. Thus, the microenvironment of the ulnar nerve sulcus is improved. The surgical incision is small, with fast recovery and no damage to the ulnar nerve nutrient vessels. Compared with ulnar nerve anterior transposition, the efficacy of treatment is significantly improved.

For patients with ulnar nerve dislocation or hypermobility, simple ulnar neurolysis may not effectively treat the underlying source of nerve irritation, which is the translation across the medial epicondyle. Tsujino *et al* reported that cubital tunnel/ulnar nerve sulcus expansion achieved good results in patients with ulnar nerve dislocation (19). Patients with ulnar nerve dislocation or hypermobility should be classified as type IV according to the criteria outlined in the present study as they are special cases. However, cubital tunnel expansion may not be suitable for all these patients. For example, if CT examinations reveal a relatively small medial humeral condyle, ulnar nerve anterior transposition is recommended instead.

In summary, based on previous classification systems that rely on sensation and strength, a new clinical classification of elbow tunnel syndrome has been established that adopts a CT imaging evaluation index. The new classification is reasonable, simple and practical. Therapies based on this classification are more targeted than those based on previous classifications.

## Figures and Tables

**Table I:** Gu’s classification of cubital tunnel syndrome (7).

Classification	Sensation	Movement	Claw-shaped hands	EMG^a^, m/sec	Treatment
Mild	Intermittent vibration paresthesia	Conscious weakness, poor flexibility	−	>40	Conservative
Moderate	Intermittent tingling paresthesia	Weak grip strength, finger adduction and abduction confined	−	40-30	Decompression
Severe	Persistent paresthesia, 2-PD abnormal	Muscle atrophy, failure of the fingers to adduct and abduct	+	<30	Anterior transposition

a Ulnar nerve conduction velocity.

EMG, electromyography; 2-PD, two-point discrimination.

**Table II:** Cubital tunnel depths, widths and depth/width ratios at various angles in the left and right elbows of normal adults (mean ± SD).

	0°			15°			30°		
			
Group	Depth, mm	Width, mm	Depth/width	Depth, mm	Width, mm	Depth/width	Depth, mm	Width, mm	Cubital tunnel index
Left elbow	1.673±0.819	12.537±1.529	0.129±0.047	2.576±0.803	13.468±2.510	0.195±0.059	3.635±0.960	13.503±2.135	0.269±0.051
Right elbow	1.860±0.539	13.243±2.703	0.130±0.042	2.846±0.851	13.419±2.753	0.213±0.053	3.546±0.822	13.039±2.676	0.277±0.059
t-value	0.679	1.002	0.035	1.165	0.066	1.168	0.357	0.693	0.520
P-value	>0.05	>0.05	>0.05	>0.05	>0.05	>0.05	>0.05	>0.05	>0.05

**Table III:** Classification and treatment selection for cubital tunnel syndrome.

Types	Sensation	Movement	EMG	Imaging (X ray, CT or MRI)	Cubital tunnel index^a^	Treatment
Type I	Ring and little finger numb, Tinel’s (+)	Conscious weakness, with or without action uncoordination	Normal	Normal	Normal	Movement control, rest, physiotherapy
Type II	Ring and little finger numb, Tinel’s (+)	Poor grip strength, decreased interosseous muscle strength or muscle atrophy	Motor and/or sensory nerve conduction velocity reduced	Normal	Normal	Ulnar neurolysis
Type III	Ring and little finger numb, Tinel’s (+)	Poor grip strength, decreased interosseous muscle strength or muscle atrophy	Motor and/or sensory nerve conduction velocity reduced	Osteoarthritis	Increased or decreased	Cubital tunnel expansion, ulnar nerve anterior transposition
Type IV	Ring and little finger numb, Tinel’s (+)	Conscious weakness, decreased interosseous muscle strength or muscle atrophy	Motor and/or sensory nerve conduction velocity reduced	Tumor, cysts, elbow deformity, post traumatic change	Normal	Targeted surgical treatment

a Depth/width ratio when the cross-section rotates 30° forward through the Hueter line (normal range, 0.273±0.055).

EMG, electromyography; CT, computed tomography; MRI, magnetic resonance imaging.

**Table IV:** Distribution of patients with cubital tunnel syndrome according to the new and Dellon/Gu’s classification.

Classification	Type I	Type II	Type III	Type IV^a^
Mild	27	0	59	3
Moderate	0	35	156	7
Severe	0	14	38	2

a Among type IV cases, there were three cases of elbow valgus deformity, five cases of ulnar nerve adhesion following elbow trauma, three cases of elbow cysts and one case of humeral condyle bone cyst.
